# Implication of Ceramide, Ceramide 1-Phosphate and Sphingosine 1-Phosphate in Tumorigenesis

**Published:** 2008-04-10

**Authors:** Patricia Gangoiti, Maria H. Granado, Alicia Alonso, Félix M. Goñi, Antonio Gómez-Muñoz

**Affiliations:** 1 Department of Biochemistry and Molecular Biology. Faculty of Science and Technology. University of the Basque Country. P.O. Box 644. 48080 - Bilbao (Spain); 2 Unidad de Biofísica (CSIC-UPV/EHU), Campus Universitario de Leioa. Barrio Sarriena s/n 48940 - Leioa (Spain)

**Keywords:** apoptosis, cell growth, ceramide, ceramide 1-phosphate, sphingosine 1-phosphate, sphingolipids

## Abstract

In the last two decades there has been considerable progress in our understanding of the role of sphingolipids in controlling signal transduction processes, particularly in the mechanisms leading to regulation of cell growth and death. Ceramide is a well-characterized sphingolipid metabolite and second messenger that can be produced by cancer cells in response to a variety of stimuli, including therapeutic drugs, leading to cell cycle arrest and apoptosis. Although this is a promising aspect when thinking of treating cancer, it should be borne in mind that ceramide production may not always be a growth inhibitory or pro-apoptotic signal. In fact, ceramide can be readily converted to sphingosine 1-phosphate (S1P) by the concerted actions of ceramidases and sphingosine kinases, or to ceramide 1-phosphate (C1P) by the action of ceramide kinase. In general, S1P and C1P have opposing effects to ceramide, acting as pro-survival or mitogenic signals in most cell types. This review will address our current understanding of the many roles of ceramide, S1P and C1P in the regulation of cell growth and survival with special emphasis to the emerging role of these molecules and their metabolizing enzymes in controlling tumor progression and metastasis.

## Introduction

**Figure 1:**
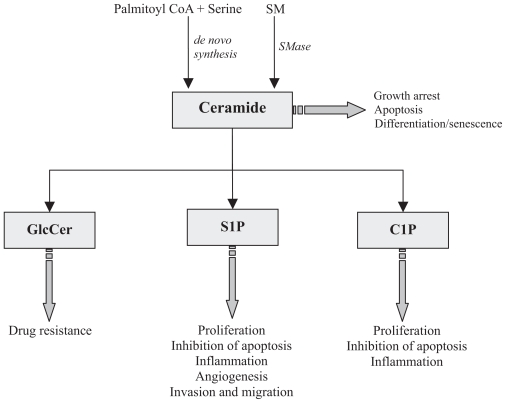
Biological roles of some sphingolipids involved tumorigenesis and metastasis. A variety of stimuli including cytokines, ionizing radiation, chemotherapeutic agents, or stress can induce ceramide formation in cells. There are two major pathways for ceramide generation: the *de novo* synthesis pathway and the hydrolysis of sphingomyelinase (SM) by sphingomyelinases (SMases). In general, ceramides mediate pro-apoptotic and anti- proliferative responses whereas sphingosine 1-phosphate (S1P) and ceramide 1-phosphate (C1P) are mitogenic and antiapoptotic for most cell types. The mechanism whereby C1P blocks apoptosis involves direct inhibition of acid SMase. Formation of glycosylceramide (GlcCer) confers resistance to a variety of drugs used in cancer therapy.

Sphingolipid metabolites have emerged as important regulators of cell biology, and some of them are implicated in the control of diverse pathophysiological processes (Merrill and Jones, 1990; Hannun, 1994, 1995, 2002; Kolesnick, 1994a, 1994b, 2002; Spiegel and Merrill, 1996; Spiegel et al. 2002, 2003, 2006; Taha et al. 2006). Sphingomyelin (SM) is one of the most important lipids in eukaryotic cells because it is the precursor of bioregulatory molecules that are capable of controlling vital cell processes. The demonstration that acid and neutral sphingomyelinases (A-SMase, and N-SMase) were activated by diacylglycerol (Kolesnick et al. 1987), or vitamin D3 (Okazaki et al. 1989), suggested that SM-derived metabolites could play relevant roles in cellular functions. These observations were reported independently by Hannun’s and Kolesnick’s groups who discovered the sphingomyelinase pathway and the physiological roles of its product, ceramide (Kolesnick, 1994; Hannun, 1994; Hannun and Obeid, 1995; Okazaki et al. 1990; Mathias and Kolesnick, 1993). In addition to regulating cell functions, ceramide is the precursor of other bioactive lipids including sphingosine-1-phosphate (S1P) and ceramide-1-phosphate (C1P), both of which are important regulators of cell signaling and metabolism in mammalian cells (Fig. 1). Another major pathway for generation of ceramides is the *de novo* synthesis pathway. This mechanism involves the sequential action of serine palmitoyltransferase (SPT), (dihydro)ceramide synthase, and (dihydro)ceramide desaturase. The latter is also the major pathway for production of complex sphingolipids, including SM and glycosphingolipids in eukaryotic cells. Recently, ceramide and some of its derivatives have been incorporated into strategies for anticancer therapies as discussed below and in recent reviews (Segui et al. 2006; Modrak et al. 2006; Reynolds et al. 2004). In normal tissues, the major component of ceramide is sphingenine, while ceramides from tumor cells contain, in addition to sphingenine, significant amounts of sphinganine (Rylova et al. 1998). Ceramide metabolism can also generate molecules with antagonistic effects. In particular, C1P and S1P can counteract many of the effects of ceramides, and conversely ceramides have opposing effects to C1P and S1P (Gomez-Muñoz, 1998, 2004, 2006). This is an important aspect because ceramide, C1P and S1P can be rapidly interconverted and this may determine the overall signal that is finally transmitted in cells. Therefore, the balance between the levels of these metabolites seems to be crucial for cell and tissue homeostasis. Switching this balance towards accumulation of S1P and C1P versus ceramide may result in abnormal stimulation of cell proliferation and/or inhibition of apoptosis leading to tumor formation. Obviously, the activity of the enzymes that are involved in the regulation of S1P, C1P and ceramide metabolism must be efficiently coordinated to ensure appropriate intracellular concentrations of these metabolites and normal cell physiology.

Many reports have shown that S1P is mitogenic and can inhibit cell death in numerous cell types (Spiegel et al. 1993, 1996, 2000a,b, 2002, 2003). Another major metabolite of ceramide is ceramide-1-phosphate (C1P), which can be formed through direct phosphorylation of ceramide by ceramide kinase. There are only a few studies suggesting that C1P is an important regulator of cell homeostasis (reviewed in Gómez-Muñoz, 2004, 2006). Increasing evidence indicates that C1P can regulate cell proliferation and that it is a potent inhibitor of apoptosis (Gómez-Muñoz et al. 1995, 1997, 2004, 2005; Mitra et al. 2007). In addition, Chalfant and co-workers have demonstrated that C1P is a positive regulator of inflammatory responses (reviewed in Chalfant and Spiegel, 2005; Lamour and Chalfant, 2005). Other reports have implicated C1P in the control of phagocytosis (Hinkovska-Galcheva et al. 1998, 2005). In the present article we review recent developments related to the control of cell homeostasis by ceramide, S1P and C1P with special emphasis to the role of these metabolites in tumorigenesis and metastasis.

## Regulation of Cell Homeostasis by Sphingolipids

Tissue homeostasis in higher organisms is determined by a network of complex processes that are tightly regulated. These include systems to balance cell proliferation and cell death to ensure proper development of the organism. Any alteration of this balance can potentially lead to disease, including autoimmune diseases, and cancer (Zhang and Xu, 2002; Vaux and Korsmeyer, 1999; Thompson, 1995). Therefore, identification of effector molecules that are involved in the regulation of cell proliferation and death is crucial for developing therapeutical strategies for prevention or treatment of these illnesses.

As mentioned above, SMases (E.C. 3.1.4.12) are key enzymes in sphingolipid metabolism because they can generate ceramide, a death signal for most cells, or C1P and S1P, which have growth factor properties. SMases are a class of enzymes that belong to the phosphodiesterase superfamily. Mammalian cells utilize three distinct forms of SMases, which can be discriminated *in vitro* by their optima pH: acidic, neutral and alkaline sphingomyelinases. Both, acidic SMase (A-SMase) and neutral SMase (N-SMase) are known to be involved in signal transduction in mammalian cells, whereas alkaline SMase is mainly responsible for digestion of dietary SM in the intestine (reviewed in Gómez-Muñoz, 2006b). Nonetheless, a potential implication of alkaline SMase in cell signaling processes has recently been suggested by Hertervig and co-workers (2003) who have demonstrated that alkaline SMase inhibits proliferation without inducing apoptosis in HT-29 human colon carcinoma cells. In addition, DNA sequencing identified a deletion of exon 4 in alkaline SMase of HT-29 cells, resulting in relatively low enzyme activity in these cells compared to normal cells (Wu et al. 2004). A reduction of alkaline SMase activity was also found in the stools of colorectal cancer patients compared to normal individuals (reviewed by Duan, 2006). For detailed information on the enzymology and compartmentalization of SMases, the reader is referred to previous reviews (Goñi and Alonso, 2002, 2006; Cremesti et al. 2002; Kolesnick et al. 2002). Concerning neutral and acidic SMases, there is evidence suggesting that stimulation of these enzyme activities is an important factor in cell cycle arrest and apoptosis (Kolesnick and Golde, 1994; Hannun, 1994; Hannun and Obeid, 2002; Hundal et al. 2003; Gomez-Munoz et al. 2003, 2004). Stimulation of SMase activity is the predominant pathway for ceramide generation in response to chemotherapy (cysplatin, doxorubicin, paclitaxel, and histone deacetylase inhibitors). Among all of the SMases, the best characterized enzyme is the acidic isoform A-SMase. In humans, mutation of the SMPD1 gene results in deficiency of A-SMase activity leading to Niemann-Pick disease types A and B, a highly lethal illness. Although A-SMase was the first SMase to be purified and cloned (Schuchman et al. 1991), regulation of its activity has only recently begun to be elucidated. Recent work by Hannun’s group has shown that A-SMase is regulated by phosphorylation through activation of PKCδ (Zeidan and Hannun, 2007), and more recently we have demonstrated that A-SMase can be regulated by the action of G_i_ proteins (Wang et al. 2007). Specifically, the bacterial toxin of *Bordetella pertussis* (pertussis toxin: PTX), which works through ADP-ribosylation of G_i_ proteins causing their inactivation, potently inhibited A-SMase and ceramide formation. By contrast, mastoparan, a well-known G_i_ protein activator, stimulated A-SMase and ceramide generation leading to apoptosis. Of importance, A-SMase can be directly inhibited by C1P, an action that involves this phosphosphingolipid in the inhibition of apoptosis and promotion of cell survival (see below).

Ceramide levels can also be controlled by SM synthase, the enzyme that catalyzes the transfer of phosphocholine from phosphatidylcholine to ceramide producing diacylglycerol and SM. In fact, this enzyme has been implicated in cell regulation and transformation through its ability to modulate the levels of ceramide and diacylglycerol, two essential bioactive lipids, and it may account, at least in part, for some of the effects previously attributed to phosphatidylcholine-specific phospholipase C (Luberto and Hannun, 1998). In addition, sphingosylphosphorylcholine (SPC), the N-deacylated form of SM, is produced under physiological and pathological conditions, and has been shown to be a positive regulator of wound healing, angiogenesis and cell proliferation, as well as causing inhibition of cell growth (Gomez-Munoz, 1998; Xu, 2002). SPC can be metabolized to sphingosine by neutral SMases (Miura et al. 2004), or to S1P by autotaxin (Clair et al. 2003). Therefore, SM deacylase may also be an important enzyme in controlling cell homeostasis.

## Ceramides and the Suppression of Tumor Progression

The direct product of SM, ceramide (N-acylsphingosine), is among the least polar, more hydrophobic lipids in cell membranes. Its solubility in water is negligible; thus, free ceramide cannot exist in solution in biological fluids or in the cytosol. A number of studies have been carried out to try to understand the behaviour of ceramides as they occur in lipid bilayers, which essentially consist of a phospholipid matrix. In systems at equilibrium, long chain (natural) ceramides have three main effects on phospholipid bilayers: a) they can increase the order of the acyl chains in bilayers, b) they give rise to in-plane phase separation of ceramide-rich and—poor domains, and c) they facilitate the transition from bilayer to non-bilayer structures (Kolesnick et al. 2000). Because of their high hydrophobicity, ceramides can exert their primary actions at the membrane level; they can only act on membrane-related proteins, that is, proteins permanently or transiently linked to membranes. In principle, this may occur in two different ways: 1) on proteins lacking specific ceramide binding sites, ceramide may act by inducing (localized) changes in the physical properties of the membrane bilayer, for example, increase in lipid chain order, which can in turn modify enzyme activities. Integral, or intrinsic proteins, are particularly sensitive to changes in bilayer order and/or fluidity (Watts, 1993). Membrane proteins may also respond to the tendency of the membranes to form non-bilayer phases, due to the presence of certain lipids that favor the lamellar-to-nonlamellar transition, for example diacylglycerol and ceramide (Epand, 1985; Kinnunen, 1996). Additional ceramide-induced changes in bilayer properties that could influence the activity of proteins not necessarily containing ceramide-binding sites, are the lateral segregation of ceramide into certain membrane domains (lipid rafts or platforms), and the ceramide-induced facilitation of protein-protein contacts; 2) ceramides may bind to specific sites in the target protein, thereby modulating enzyme action. This mechanism may operate not only on proteins that are permanently bound to membranes, but also protein molecules transiently recruited to the bilayer where ceramides are located (see Kolesnick et al. 2000; Goñi and Alonso, 2006 for further details). There are two groups of proteins that are known to contain ceramide-binding sites: a) proteins that are transiently bound to membranes, such as ceramide-activated protein kinase (CAPK), some protein kinase C (PKC) isoforms, or c-Raf-1; b) proteins without known membrane-binding capacity, such as ceramide-activated protein phosphatase 1 (CAPP1) or ceramide-activated protein phosphatase 2 (CAPP2A) CAPPs are among the best characterized ceramide targets. Treatment with exogenous ceramide or TNFα, a ceramide-generating agonist, caused dephosphorylation of c-Jun in A431 cells. Pre-treatment of these cells with okadaic acid completely blocked the effects of exogenous ceramide and TNFα on c-Jun, suggesting a PP2A form of CAPP (Reyes et al. 1996). Increased mitochondrial PP2A activity and specific dephosphorylation of mitochondrial Bcl-2 in response to ceramide has also been demonstrated (Ruvolo et al. 1999; Ruvolo et al. 1998). Another described substrate for CAPP is PKB (Akt). This kinase mediates many anti-apoptotic biological actions (Sato et al. 2000) and plays key roles in insulin action and in mitogenic signaling (Hajduch et al. 2001). PKB has been demonstrated to be dephosphorylated by PP2A prior to activation of caspase 9 and Bad (Basu et al. 1998; Cardone et al. 1998) and has been suggested to be a necessary event in turning off a key anti-apoptotic mechanism. Several lines of evidence have indicated the ability of ceramide to induce dephosphorylation of PKB with concomitant loss of function/activity (Reviewed by Pettus et al. 2002), and pre-treatment with okadaic acid revealed a role for PP2A in regulating the activity of this enzyme (Hajduch et al. 2001; Cazzolli et al. 2001; Schmitz-Peiffer et al. 1999; Salinas et al. 2000). More recently, activation of CAPP has been negatively implicated in the regulation of PKC autophosphorylation, leading to inactivation of this enzyme activity. The specific isoforms that were sensitive to dephosphorylation by CAPP were PKC α and βII (Kitatani et al. 2007). In particular, activation of these PKC isoforms leads to stimulation of phospholipase D (PLD), an enzyme that produces promitogenic phosphatidic acid (PA) (reviewed by Exton, 1999). Indeed, we previously demonstrated that short-chain ceramides are potent inhibitors of PLD in different cell types (Gomez-Muñoz et al. 1994, 1995, 2000, 2001; Pérez-Andres et al. 2002), an action that supports the antiproliferative effect of ceramides.

An important aspect of ceramide action concerns its transport from the ER, where it is synthesized *de novo*, to the Golgi apparatus, the primary site of SM and glycosphingolipid synthesis. In this concern, Hanada and co-workers demonstrated the existence of a specific protein that is crucial for SM biosynthesis and acts as a ceramide transfer protein (CERT) in a non-vesicular manner. This protein has two suitable domains for the transport of ceramide: one domain that recognizes ceramide and mediates its intermembrane transfer, termed the START domain, and a phosphatidylinositol binding domain (PH domain) with selectivity towards phosphatidylinositol-4-phosphate, a lipid that is enriched in the Golgi and that could serve as the site for ceramide delivery by CERT (Hanada et al. 2003).

Ceramides have been shown to be mediators of cellular stress. There are many agents that can induce accumulation of ceramides within cells; these include cytokines (TNFα, interleukins, FasL), UV or ionizing radiation, heat shock, hypoxia, or chemotherapeutic drugs such as daunorubicin, vincristine, etoposide, fenretidine, cysplatin, among others. The biological activity of ceramides is influenced by different structural elements. In particular, insertion of a trans 4–5 double bond in the molecule, an action that is catalyzed by (dihydro)ceramide desaturase, seems to be crucial for ceramide activity; also, ceramides can be more or less active depending on the length of the N-acyl chain that is linked to the sphingosine backbone (Goñi and Alonso, 2006; Modrak et al. 2006). Similarly, sphingomyelins with differing N-acyl chain lengths are capable of providing varying degrees of chemosensitization (Modrak et al. 2006). The kinetics of ceramide formation in response to different stimuli are complex and variable; this responses range from seconds to hours, and the same stimulus can generate different responses in different cell types (Hannun, 1996). Unexpectedly, it was observed that ceramide levels were higher in ras or tyrosine kinase ( *fps*) transformed fibroblasts compared to non-transformed cells (Martin et al. 1997), and similar results were obtained in human head and neck squamous cell carcinomas (HNSCC) compared to noncancerous tissues (Koybasi et al. 2004); however, when individual ceramide species were examined the results demonstrated that only C18:0-ceramide was lower in HNSCC tumor tissues as compared to their noncancerous counterparts (206 versus 392 pmol/mg protein, respectively) whereas the levels of all the other ceramide species were generally higher in HNSCC tumors than in their controls. It was concluded that C18:0-ceramide plays a role in the pathogenesis/progression of the HNSCC (Koybasi et al. 2004). It was also reported that ceramides are inversely associated with malignant progression of human astrocytomas, which supports the rational for the potential benefits of ceramide-based chemotherapy (Riboni et al. 2002b).

### Molecular targets of ceramides

The mechanisms whereby ceramides exert their biological actions include modulation of diverse signal transduction pathways and key regulatory enzymes. CAPK is a member of a family of proline-directed serine/threonine kinases (Liu et al. 1994), whose activity can be enhanced by treatment of intact cells with short-chain ceramide analogs, bacterial sphingomyelinases, TNFα, or interleukin 1-beta (IL-1β) (Zhang and Kolesnick 1995). CAPK was identified as the kinase suppressor of Ras (KSR), and was associated with phosphorylation and activation of Raf-1 and the subsequent activation of MAPK (Yao et al. 1995; Zhang et al. 1997). As previously mentioned another well-defined target for ceramide action is CAPP (CAPP1 and CAPP2A) (Dobrowsky and Hannun, 1992; Kolesnick, 2002). Some specific substrates of CAPP have already been identified; these include c-Jun (Reyes et al. 1996), Bcl-2 (Ruvolo et al. 1999), protein kinase B (PKB) (Zhou et al. 1998), PKC-α (Lee et al. 1996), retinoblastoma protein (Rb) (Plummer et al. 2005) and SR proteins (Chalfant et al. 2001). Ceramides also can directly activate the protein kinase C isoform PKC-ζ (Lozano et al. 1994; Muller et al. 1995), leading to stimulation of stress-activated protein kinases (SAPK) also known as Jun nuclear kinases (JNKs). Activation of this pathway leads to multiple effects, including alternative splicing through activation of hnRNP A1 and inhibition of protein synthesis via the RAX-PKR (double-stranded RNA-dependent protein kinase/elIF1-alpha pathway (Modrack et al. 2006). Thus, the protease cathepsin D is activated by ceramides and participates in the activation of executionary caspase 3 after translocation from the lisosomes (De Stefanis et al. 2002). Also, inhibition of the nuclear transcription factor NF-κB by ceramides has been observed in a variety of cell systems (reviewed by Gómez-Muñoz, 1998, 2004, 2006). Lastly, another important target of ceramide is phospholipase D (PLD). It was first demonstrated that ceramides are potent inhibitors of PLD activity in rat fibroblasts that were challenged with different agonists including mitogenic agents such as lysophosphatidic acid (LPA), S1P, thrombin, or serum (Gómez-Muñoz etal. 1994, 1995). Ceramides were also able to block PLD activation by oxidized low density lipoproteins (Gómez-Muñoz et al. 2000), which were shown to stimulate growth of murine macrophages (Hundal et al. 2003). The inhibitory effect of ceramide on PLD is likely to be caused by direct interaction of the sphingolipid with enzymes or factors that directly activate PLD, such as PKCα or the small G proteins ADP ribosylation factor (ARF) and Rho. Therefore, in addition to inducing apoptosis, ceramides are negative regulators of signalling pathways that are associated with the stimulation of cell proliferation (Gómez-Muñoz, 1998).

As mentioned above, formation of ceramide is equally relevant because it is the precursor of important bioactive sphingolipids that can also regulate cellular functions (Fig. 1). Hence, stimulation of ceramidases results in generation of sphingosine, which was first described as an inhibitor of protein kinase C (PKC) (Hannun et al. 1986) and can also inhibit phosphatidate phosphohydrolase (PAP) activities (Gómez-Muñoz et al. 1992). These actions may influence cell homeostasis because diacylglycerol, the product of PAP activity, and PKC are positive regulators of cell proliferation. Sphingosine, in turn, can be phosphorylated by the action of sphingosine kinases to generate S1P, which is a potent mitogenic agent and can also inhibit apoptosis in many cell types (Wu et al. 1995; Spiegel and Merrill, 1996; Spiegel et al. 1993, 1996; Spiegel and Milstein, 2002, 2003; Hait et al. 2006; Taha et al. 2006). Thus, ceramidases might be important enzymes for tumor promotion. In this connection, human acid ceramidase has been shown to be overexpressed, although not mutated, in 42% prostate cancer specimens and in three prostate cancer cell lines (Seelan et al. 2000) and inhibition of ceramidase activity with aromatic ceramide analogues showed effective anticancer activity in melanoma and colon cancer cells (Raisova et al. 2002; Selzner et al. 2001).

### Therapeutic possibilities of ceramide metabolism

Chemotherapy-mediated increases of ceramide levels in tumor cells may be due to stimulation of the *de novo* synthesis pathway, to an increased SMase activity, or to disruption of ceramide catabolism. Therefore, manipulation of the pathways responsible for ceramide formation and its metabolism may be effective targets for cancer therapy. Well-defined targets in the *de novo* synthesis pathway are serinepalmitoyl transferase (SPT), of which there are two isoforms, SPT1 and SPT2, and ceramide synthase activities. Induction of these enzymes would lead to an increase in ceramide levels. In particular, the synthetic anti-cancer drug fenretinide increases the activity of both SPT and ceramide synthase, leading to growth inhibition of neuroblastoma, melanoma, and other tumors (reviewed by Ribatti et al. 2003). Paclitaxel, or etoposide can also elevate ceramide levels through stimulation of the *de novo* pathway, and anthracylines like doxorubicin can stimulate both the SMase and the *de novo* synthesis pathways. Paradoxically, SPT expression has been shown to be enhanced in proliferating fibroblasts, some transformed cell lines and some human tumors (Batheja et al. 2003). In the latter report both SPT1 and SPT2 appeared to be associated with the nucleus in actively proliferating cells, whereas the two SPT isoforms were much less abundant in nuclei of quiescent cells. The function of SPT1 and SPT2 associating with the nucleus in de-differentiated, proliferating cells is unknown at present, but it might be possible that nuclear localization of SPT is implicated in sphingolipid nuclear signaling. The contradictory observation that SPT can be implicated in promoting apoptosis as well as cell proliferation could be explained by the different levels of expression of ceramide kinase, and/or SK in different cell types. Cells with low levels of ceramide kinase or SK activities will have difficulties to convert SPT-derived proapoptotic ceramide into antiapoptotic or promitogenic C1P or S1P, whereas cells with high levels of these kinases will rapidly form C1P and S1P at the expense of ceramides. Therefore, another possibility for improving cancer therapy would be to prevent the metabolism of ceramide to S1P or C1P, as these metabolites have opposing effects to ceramide. In this context, drugs like the inhibitor B13, which potently blocks ceramidase activity, have been shown to enhance tumor cell apoptosis (Selzner et al. 2001). Also, safingol (L-threo-dihydrosphingosine), a potent sphingosine kinase (SK) inhibitor, is commonly used in cancer therapy to avoid formation of S1P, and the recently identified inhibitor of ceramide kinase, F-1209A, can be used to inhibit C1P generation (Kim et al. 2005).

Ceramidase-derived sphingosine may also be acylated by ceramide synthase to form a stereochemically variant L-threo ceramide that cannot be glucosylated by glycosylceramide synthase (GCS) to generate glycosylceramide (reviewed by Reynolds et al. 2004) and this has been shown to synergistically enhance tumor cell killing by the ceramide-generating retinoid fenretidine (Maurer et al. 2000). The latter compound, also known as N-(4-hydroxyphenyl) retinamide, is cytotoxic for human leukemia cells through generation of ceramides by both SMase activation and stimulation of the *de novo* pathway (Morales et al. 2007). Robust ceramide generation through activation of the *de novo* pathway was also observed after administration of the natural phytochemical curcumin to human colon cancer cells, and this led to cell death by apoptosis (Moussavi et al. 2006). Improvement of cancer therapy could also be achieved by preventing glycosphingolipid formation after administration of ceramide-producing drugs. Glycosylceramide (GlcCer), lactosylceramide, and gangliosides play essential roles in cell development, cell death, and tumor progression. Radioresistant melanoma cells rich in gangliosides can be made radiosensitive by exposure to fumonisin B1, which blocks ganglioside biosynthesis at the level of ceramide synthase. Conversely, adding bovine brain ganglioside M1 to radiosensitive melanoma cells can confer radioresistance. Several reports have shown that over-expression of glucosylceramide synthase (GCS), or P-glycoprotein transporters induce drug-resistance. This suggests that shunting ceramide to a glycosylated form may decrease the cytotoxic effects of drug-producing ceramides, thus inhibition of this step might potentiate toxicity of chemotherapeutic agents thereby improving cancer cell killing. A comprehensive review by Gouaze-Andersson and Cabot, (2006), discusses the role of glycosphingolipids in drug resistance toward cancer treatment.

## Sphingosine-1-Phosphate and Ceramide 1-Phosphate Promote Cell Growth and Survival—Possible Role in Tumorigenesis

Stimulation of ceramidases generates sphingosine, which can control the activity of key enzymes involved in the regulation of metabolic or cell signaling pathways, i.e. inhibition of the Mg^2+^ dependent form of phosphatidate phosphohydrolase (PAP) (Jamal et al. 1991; Gómez-Muñoz et al. 1992), inhibition of PKC (Hannun et al. 1986), activation of PLD (Natarajan et al. 1994), or stimulation of diacylglycerol kinase (Sakane et al. 1989; Yamada et al. 1993). However, many of the effects of sphingosine are known to be mediated through its conversion to S1P (Desai et al. 1992). In addition to being produced intracellularly, many cells can secrete important amounts of S1P under certain circumstances. S1P is present in serum at relatively high concentrations (up to 1 μM), where it is bound to albumin. It is mainly produced and stored in red blood cells and platelets, and it can be released to the extracellular milieu upon cell activation (Pappu et al. 2007). Extracellular S1P can also be secreted by other cell types such as mast cells and monocytes (Watterson et al. 2003). The mechanisms that regulate S1P secretion are poorly understood at the present time. The intracellular levels of S1P are under strict regulation by the enzymes that control its biosynthesis and degradation. Sphingosine kinase (SK) catalyzes the ATP-dependent phosphorylation of sphingosine to generate S1P, whereas degradation of S1P can be mediated by two different pathways: a) the reversible dephosphorylation to sphingosine by different phosphatases, and b) the irreversible degradation to hexadecenal and ethanolamine phosphate by specific lyase activity (Merrill and Jones, 1990). SK activity has been demonstrated in most mammalian tissues, yeast, and plants. In mammals, two distinct SK isozymes (termed SK1 and SK2) have been characterized. These two isoforms have different kinetic properties and are differently distributed among tissues suggesting that they regulate different cellular functions. In particular, SK1 has been shown to promote cell survival, proliferation, and tumor cell growth (Johnson et al. 2002; Nava et al. 2002; Taha et al. 2004, 2006; Kohno et al. 2005), whereas SK2 has been described as a putative BH3-only protein that is associated with the induction of apoptosis, and cell cycle arrest (Liu et al. 2003; Maceyka et al. 2005). Stimulation of SK activity has been observed in response to a variety of agonists including TNF-α, nerve growth factor (NGF), vascular endothelial growth factor (VEGF), platelet-derived growth factor (PDFG), epidermal growth factor (EGF), or basic fibroblast growth factor (reviewed by Saba and Hla, 2004). The mechanisms whereby SK activity is stimulated have not been fully described, but it is known that SK1 is phosphorylated by protein kinase C (PKC) upon treatment of endothelial cells with VEGF (Rosenfeldt et al. 2001) and that SK1 gene expression is mediated by PKC- and ERK-dependent signal transduction pathways (Spiegel and Milstein, 2003).

The degradation of S1P can be catalyzed by different phosphatases, and by S1P lyase (Brindley and Waggoner, 1996; Giussani et al. 2006; Saba and Hla, 2004; Merrill and Jones, 1990). There are two isoforms of S1P phosphatase (SPP), which are designated SPP1 and SPP2. Both of these phosphatases localize to the endoplasmic reticulum (Saba and Hla, 2004) where they dephosphorylate S1P specifically. In addition, S1P can be dephosphorylated by a Mg^2+^-independent and N-ethylmaleimide insensitive phosphatidate phosphohydrolase (PAP-2) that resides in the plasma membrane of cells (Waggoner et al. 1996). PAP-2 belongs to a family of enzymes that were renamed lipid phosphate phosphatases (LPPs) (Brindley and Waggoner, 1996). This new nomenclature was adopted because this enzyme can also catalyze the dephosphorylation of various other phospholipids besides phosphatidic acid (PA), mainly lysoPA, C1P, and S1P. SPPs differ from LPPs in that SPPs are highly specific toward long chain sphingoid base phosphates; also the enzymological properties of these two families of phosphatases are different (Le Stunff, 2002; Ogawa et al. 2003). As for the role of mammalian SPPs it was found that dephosphorylation of S1P stimulated the *de novo* synthesis of ceramide (Le Stunff et al. 2004). This observation is consistent with the stimulation of PAP-2 by C_2_-ceramide that we previously reported (Gómez-Muñoz et al. 1994), and with the ability of this cell-permeable ceramide to stimulate the metabolism of S1P and the production of intracellular ceramides (Posse de Chaves et al. 1997; Gómez-Muñoz et al. 1995; Ogretmen et al. 2002). More recently, SPP1 was shown to also regulate the ER-to-Golgi trafficking of ceramide (Giussani et al. 2006), and SPP2 has been demonstrated to be induced during inflammatory responses in different cell types (neutrophils, endothelial cells, and epithelial cells (Mechtcheriakova et al. 2007). Increases in SPP2 activity occurred at late time points after treatment with various agonists. TNF-α, Lipopolysaccharide (LPS), or the phorbol ester PMA increased mRNA levels of SPP2, after 6–8 h treatment, whereas none of these agonists or the growth factor EGF caused any significant changes in SPP2 activity at early time points (Mechtcheriakova et al. 2007). The latter authors also demonstrated that the SPP2 promoter has two nuclear factor-kappa B (NF-κB) binding sites. Specifically, Rel A is the NF-κB subunit that is critical for SPP2 induction of transcription. The importance of SPP1 as a key regulatory enzyme in cell homeostasis has been recently highlighted by the discovery that overexpression of this enzyme in human embryonic kidney cells suppresses motility towards epidermal growth factor (EGF) by reducing S1P accumulation in response to EGF. In agreement with this, downregulation of SPP1 increases the intracellular levels of S1P, and facilitates its secretion to the extracellular milieu. This action results in activation of a heterotrimeric Gi protein-coupled S1P receptor, possibly by an autocrine pathway (Le Stunff et al. 2004), and has important implications on cell viability (Johnson et al. 2003). It can be concluded that dephosphorylation of S1P can be catalyzed by both specific SPPs, and LPPs, and that these actions may have profound effects in the control of cell growth and survival, as well as in inflammation.

The irreversible degradation of S1P is catalyzed by S1P lyase through cleavage of the C2–3 carbon bond of S1P. This generates palmitaldehyde and ethanolamine phosphate (Merrill and Jones, 1990). Like SPPs, S1P lyase is found in the ER (Saba and Hla, 2004) where most of the essential enzymes for sphingolipid biosynthesis are localized. It has been shown that S1P lyase stimulates the *de novo* synthesis of ceramides, and that induces apoptosis through stimulation of the intrinsic apoptotic pathway that is associated to cytochrome c release from mitochondria (Reiss et al. 2004). It was postulated that S1P lyase can regulate mammalian cell survival by controlling the intracellular levels of S1P and ceramides (Reiss et al. 2004; Saba and Hla, 2004). Also, it was reported that S1P lyase is implicated in the regulation of important biological functions in different organisms. For instance, in *C. elegans*, *Dictyostelium*, or *Drosophila*, this enzyme activity plays an important role in embryogenesis, reproduction, survival, or movement (Saba and Hla, 2004). More recently, S1P lyase has been shown to potentiate apoptosis via p53 and p38-dependent pathways, and that it is downregulated in colorectal carcinomas (Oskouian et al. 2006). These observations led to the prediction that loss of S1P lyase expression could contribute to tumor cell survival. In fact, deletions of the 10q21 chromosomal region where the human S1P lyase locus resides have been demonstrated in a variety of cancers (Ichimura et al. 1998; Petersen et al. 2000; Petersen et al. 1998). Basal intracellular concentrations of S1P is in the range of low pmols per mg of protein (about 2.3 pmol/mg protein in Chinese Hamster Ovary cells ) (Tani et al. 2005) and this amount is to be higher in tumor cells lacking S1P lyase.

Another putative tumor-promoting sphingolipid is C1P. The existence of C1P was first reported by Dressler and Kolesnick in human leukemia (HL-60) cells (Dressler and Kolesnick, 1990). Ceramide kinase (CERK), the enzyme responsible for C1P formation in cells, was first identified in brain synaptic vesicles by Bajjalieh and co-workers (1989), and then found in human leukemia HL-60 cells (Kolesnick and Hemer, 1990). This enzyme was confined to the microsomal membrane fraction, and it phosphorylated ceramide in the presence of physiological calcium concentrations. Of importance, human CERK has been recently cloned by Sugiura and co-workers (Sugiura et al. 2002). With regard to substrate specificity, it was reported that phosphorylation of ceramide by CERK is stereospecific (Wijesinghe et al. 2005). The latter report also showed that a minimum of a 12-carbon acyl chain was required for normal CERK activity. The importance of CERK in cell signaling was highlighted using specific siRNA to downregulate this enzyme activity in A549 lung adenocarcinoma cells. This treatment dramatically inhibited arachidonic acid release and prostaglandin E_2_ (PGE_2_) production in response to ATP, the calcium ionophore A23187 and interleukin 1-β (Chalfant and Spiegel, 2005; Pettus et al. 2003). It has been known for a long time that there is a strong association between chronic inflammation and cancer. The fact that C1P is a strong activator of cPLA_2_ places CERK as a new potential therapeutic target for cancer treatment and/or prevention.

Recently, a human ceramide kinase-like (CERKL) enzyme was identified in retina (Tuson et al. 2004) and subsequently cloned (Bornancin et al. 2005). However, this enzyme was unable to phosphorylate ceramide, or other related lipids, under conditions commonly used to measure CERK activity, and therefore its role in cell biology is unclear.

It was previously reported that C1P can be formed in neutrophils upon addition of exogenous cell-permeable [^3^H]*N*-hexanoylsphingosine (C_6_-ceramide) to the cells (Rile et al. 2003). Riboni and co-workers (2002a) demonstrated that C1P could be generated in cerebellar granule cells both from SM-derived ceramide and through the recycling of sphingosine produced by ganglioside catabolism. C1P can be also generated by the action of interleukin 1β on A549 lung adenocarcinoma cells (Pettus et al. 2003), and plays an important role in inflammation (Chalfant and Spiegel, 2005; Pettus et al. 2003, 2004, 2005; Subramanian et al. 2005). We found that C1P is present in normal bone marrow-derived macrophages (Gómez-Muñoz et al. 2004), and that its levels are substantially decreased in apoptotic macrophages. This observation suggested that C1P may play an important role in cell survival (Gómez-Muñoz, 2004b, 2006). The identification of C1P phosphatase in rat brain (Shinghal et al. 1993) and hepatocytes (Boudker and Futerman, 1993) together with the existence of CERK suggested that ceramide and C1P are interconvertible in cells. C1P phosphatase is enriched in brain synaptosomes and liver plasma membrane fractions, and appeared to be distinct from the phosphatase that hydrolyzes phosphatidic acid (PA), PA phosphohydrolase (PAP). Nonetheless, C1P can also be converted to ceramide by the action of a PAP activity that is specifically located in the plasma membrane of cells (Waggoner et al. 1996). The latter enzyme belongs to a family of at least three mammalian lipid phosphate phosphatases (LPPs) (Brindley and Waggoner, 1998). LPPs have recently been shown to regulate cell survival by controlling the levels of intracellular PA and S1P pools (Long et al. 2005) and to also regulate leukocyte infiltration and airway inflammation (Zhao et al. 2005). Dephosphorylation of C1P might be a way of terminating its biological effects, and the resulting formation of ceramide could potentially be detrimental for cells. In any case, controlling the levels of ceramide and C1P by the coordinated action of CERK and C1P phosphatases, may be of crucial importance for the metabolic or signaling pathways that are regulated by these two sphingolipids.

### Biologic effects of sphingosine-1-phosphate and ceramide 1-phosphate

The tumor microenvironment is rich in lipid metabolites, of which one of the most relevant is S1P. Receptors for bioactive lipids including S1P are involved in aberrant cell proliferation in a wide range of cancer cells. S1P was first described as a mitogen for cultured fibroblasts (Zhang et al. 1990) and was subsequently implicated in the regulation of cell migration, survival, invasion, and angiogenesis (Ancellin et al. 2002; Merrill et al. 2002; Payne et al. 2002; Donati and Bruni, 2006), processes that are all essential for tumor progression. Most pro-angiogenic factors such as vascular endothelial growth factor (VEGF), promote the translocation of SK1 to the plasma membrane, thereby promoting the local accumulation of S1P. In turn, S1P stimulates its G-protein coupled receptors (GPCRs S1P_1–5_) in several tumor and stromal cells. Among them, S1PR1, previously known as EDG-2, is highly expressed in endothelial cells. Stimulation of this receptor promotes the secretion of VEGF by endothelial cells, and inhibition of S1P with a blocking antibody prevents the release of IL6, IL8 and VEGF by tumor cells, suggesting that S1P production and S1P_1_ activation are required to elicit an angiogenic response (Dorsam and Gutking, 2007). Just as cancer cells co-opt chemokine and bioactive lipid networks to invade surrounding tissue, reach the vascular and lymphatic circulation and migrate and invade their target tissues, they can also manipulate these networks and their GPCRs to attract endothelial cells and instruct them to invade the tumor mass, thereby forming new vessels to provide nutrients and oxygen.

C1P was also found to be mitogenic as it stimulated DNA synthesis and cell division in rat fibroblasts (Gómez-Muñoz et al. 1995). The first studies were performed using synthetic short-chain C1Ps (C_2_-C1P and C_8_-C1P) that were enzymically synthesized using C_2_- and C_8_-ceramides as substrates. Of interest, C1P was also able to reverse the morphological changes that were induced in rat fibroblasts after prolonged serum deprivation, a condition that induces apoptosis in those cells (Gómez-Muñoz, 1998), suggesting a role of C1P in the promotion of cell survival. This new action of C1P was later confirmed in bone marrow-derived macrophages that were incubated in the absence of growth factors (see below).

In rat fibroblasts, the mitogenic effects of S1P and C1P could be blocked by ceramides, possibly through a mechanism involving stimulation of S1P and C1P degradation by LPP activity (Gómez-Muñoz et al. 1995a,b). S1P and C1P have been demonstrated to counteract the pro-apoptotic effect of ceramide (Olivera and Spiegel, 1993; Gómez-Muñoz et al. 2004, respectively). Therefore, ceramides and S1P or C1P play antagonistic roles in cell signaling. These findings, together with the observation that S1P regulates ceramide synthesis, support the idea of the sphingolipid “rheostat model”, in which the balance between S1P and ceramides is crucial for cell fate decisions (Spiegel and Milstein, 2003). Many of the effects of S1P are elicited through its interaction with specific Gi protein-coupled receptors (S1P_1–5_) that are ubiquitously expressed and regulate numerous downstream signals. These aspects of S1P signaling have been recently discussed in several comprehensive reviews (Watterson, 2003; Saba and Hla, 2004; Spiegel and Milstein, 2003; Payne et al. 2002; Donati and Bruni, 2006), therefore they will not be addressed in detail here. Also, S1P has been shown to be generated intracellularly by the action of certain growth factors, and serum (Olivera and Spiegel, 1993; Coroneos et al. 1995). In particular, PDGF, or serum stimulate SK activity and the production of S1P in Swiss 3T3 fibroblasts (Rani et al. 1997). However, no direct intracellular targets of S1P have so far been conclusively characterized. It has been suggested that S1P may play a role in the regulation of cell cycle progression, as PDGF can induce translocation of SK into the nuclear envelope and increase nuclear-associated SK activity (Kleuser et al. 2001). Activation of SK by PDGF leads to mitogen-activated protein kinase (MAPK) extracellular regulated kinases 1/2 (ERK 1/2) stimulation, and S1P inhibits c-Jun N-terminal kinase (JNK) activation (Cuvillier et al. 1996). The latter findings are relevant because the extent of activation of ERK versus JNK is an important aspect in the regulation of apoptosis (Xia et al. 1995). S1P has been shown to act intracellularly as a second messenger to regulate cell proliferation, suppression of apoptosis, and cell survival (Payne et al. 2002; Spiegel and Milstein, 2002; Taha et al. 2006; Donati and Bruni, 2006). The biological actions elicited by S1P involve activation of diverse signaling pathways. For example, effects of S1P on cytoskeletal rearrangement and cell motility are mediated by the small GTPases Rac and Rho, whereas stimulation of cell proliferation and cell survival by S1P involves intracellular Ca^2+^ mobilization and activation of MAPK, phospholipase D, and transcription factors such as AP-1 (Payne et al. 2002; Spiegel and Milstein, 2002). Therefore, S1P can function both as first and second messenger, and these actions may differentially affect specific intracellular targets or signaling pathways (Vann et al. 2002).

Two recent reports showed that S1P and C1P increased cell survival in bone marrow-derived macrophages that were incubated in the absence of M-CSF, a condition that induces apoptosis in these cells (Gómez-Muñoz et al. 2003). It was demonstrated that both of these phosphosphingolipids inhibited caspase 9 and 3 activation, as well as DNA fragmentation, indicating that they were blocking apoptosis. In addition, both S1P and C1P prevented the accumulation of ceramides that occurs in apoptotic macrophages incubated in the absence of M-CSF. Both acid and neutral SMase activities were upregulated by M-CSF deprivation, but nearly all of the SMase activity was attributable to the acidic form of the enzyme. A key observation in those studies was that S1P or C1P completely inhibited the activation of A-SMase in intact cells, which resulted in blockade of ceramide accumulation. It is not clear whether or not these effects of S1P and C1P are receptor-mediated events. However, experiments performed in the presence of pertussis toxin failed to reverse the inhibitory effect of these metabolites on A-SMase activation. In addition, A-SMase activation was independent of cAMP, as treatment with the adenylyl cyclase activating agent forskolin, at concentrations shown to elevate cAMP, did not alter A-SMase activity in the macrophages. These data are in agreement with previous studies suggesting that the cytoprotective effects of S1P are receptor-independent events (Spiegel and Milstein, 2003). Another key observation was that C1P blocked the activity of A-SMase in cell homogenates suggesting that inhibition of this enzyme occurs by direct physical interaction with C1P. Consequently, C1P was considered to be a selective inhibitor of A-SMase. Inhibition of this enzyme has now been demonstrated to be a major mechanism whereby C1P promotes cell survival (Gómez-Muñoz et al. 2004). This observation also suggested that inhibition of A-SMase by C1P is not mediated through receptor interaction. Interestingly and contrary to C1P, the inhibitory effect of S1P did not involve direct interaction with A-SMase. Activation of A-SMase also plays a key role in pulmonary infections as it facilitates internalization of bacteria into lung epithelial cells (Gulbins and Kolesnick, 2003). Therefore, inhibition of A-SMase by C1P or S1P could be an important mechanism to reduce or prevent infection of mammalian cells.

An important pathway that is a positive regulator of cell survival is the phosphatidylinositol 3-kinase (PI3-K)/protein kinase B (PKB) pathway. In mammalian cells, PKB (also known as Akt) is activated downstream of PI3-K when cells are stimulated by growth factors, insulin, or certain G-protein-coupled receptor agonists. Once activated, PKB can promote a variety of cellular responses, including cell growth, inhibition of apoptosis, cell proliferation, or cell migration. Many of the functions that are regulated by PKB involve binding of 14-3-3 proteins to the sites of PKB phosphorylation. Concerning apoptosis, 14-3-3 proteins have been shown to bind to YAP65, and transcription factors of the FOXO family. In addition, PKB can also positively regulate G1/S cell-cycle progression through different mechanisms, including phosphorylation of the CDK inhibitor p27 (Kip1), and 14-3-3 proteins have been shown to bind these PKB-phosphorylated sites (reviewed by Mackintosh 2004).

We previously demonstrated that activation of the PI3-K/PKB pathway, but not MAPK, was required for the anti-apoptotic effect of S1P and C1P in BMDM (Gómez-Muñoz et al. 2003, 2005). PI3-K activation was demonstrated by immunoprecipitation of the enzyme from whole cell lysates and assayed *in vitro* using ^32^P-phosphatidylinositol. An *in vivo* approach provided evidence of phosphatidylinositol (3,4,5)-trisphosphate (PIP3) formation in intact cells that were prelabeled with ^32^P-orthophosphate. PIP3 is a major product of PI3-K activity, and has recently been shown to directly inhibit A-SMase (Testai et al. 2004). It is possible that PI3-K activation might enhance the inhibitory effect of C1P or S1P on ASMase through generation of PIP3. Whether C1P and PIP3 bind to the same or different domains of A-SMase remains to be determined. The expression of anti-apoptotic Bcl-X_L_, which is regulated by PI3-K, was also decreased by M-CSF withdrawal, and S1P or C1P restored Bcl-X_L_ to normal levels. It was concluded that the PI3-K/PKB signaling cascade is a positive regulator of S1P- or C1P mediated cell survival, and that inhibition of A-SMase activity and the subsequent decrease in ceramide levels by S1P or C1P is an important factor for maintaining PKB activation and macrophage survival. In fact C_2_-ceramide, but not the inactive form dihydro- C_2_-ceramide, blocked S1P- or C1P-induced cell survival, thereby emphasizing the importance for cells to maintain an appropriate balance between the intracellular levels of these metabolites. The metabolic or signaling pathways that are regulated by C1P are not well characterized, but unlike S1P, C1P does not affect PLD, MAPK (ERK 1/2), adenylyl cyclase, Ca^2+^ mobilization, or the expression of the early genes *c-fos* or *c-myc* in rat or mouse fibroblasts (Gómez-Muñoz, 1995, 1997). Nonetheless, a slight induction of ERK phosphorylation was demonstrated in human osteoblastic cells stimulated with short-chain C1P (Carpio et al. 1999) and we have preliminary evidence suggesting that natural C1P stimulates macrophage proliferation through ERK1/2 activation (P. Gangoiti et al. unpublished work). It is obvious from the above observations that the activity of the enzymes involved in ceramide, C1P and S1P metabolism must be strictly regulated so that cells can keep appropriate levels of pro- apoptotic versus anti-apoptotic metabolites. Any alteration in the balance between ceramides, C1P and S1P could potentially result in development of illnesses including cancer.

### Targeting sphingosine 1-phosphate and ceramide 1-phosphate formation as therapeutic possibilities

Regulation of S1P and C1P metabolism provides a new therapeutic possibility for cancer treatment. The blockage of S1P and C1P formation will lead to inhibition of proliferation, as well as the induction of apoptosis in cancer cells. Recently identified SK1 inhibitors have been shown to inhibit cancer cell proliferation and tumor growth *in vitro* and *in vivo* in animal models (French et al. 2006; Ségui et al. 2006). However, specific inhibition of SK1 can be complicated, because SK1 shares with SK2 two large conserved regions, which feature about 80% similarity at the protein level, as well as five short sequence stretches, designated C1–C5. Another possibility is preventing interaction of S1P with its receptors. There are some novel S1P_1_ and S1P_3_ antagonists (Davis et al. 2005) that could inhibit cancer cell growth. However, there is evidence suggesting that some of the effects of S1P are receptor independent (Kohno et al. 2005). In addition, targeting S1P receptors might be complicated because of the existence of multiple isoforms with different responses (Ancellin et al. 1999; Spiegel and Milstien, 2000; Ségui et al. 2006).

A novel approach to cancer treatment uses a monoclonal antibody that binds S1P with high affinity and specificity. The anti-S1P mAb substantially reduced tumor progression and in some cases it eliminated measurable tumors in murine xenograft and allograft models. The anti-S1P mAb inhibited tumor associated angiogenesis, S1P- induced proliferation, and the ability of S1P to protect tumor cells from apoptosis in several tumor cell lines (Visentin et al. 2006).

In 1858, Rudolf Virchow suggested that cancers frequently occured at sites of chronic inflammation. In the light of the pro-survival and pro-inflammatory functions of C1P, CERK, the enzyme responsible for C1P formation, might be an important target for the development of novel anti-cancer drugs. In this regard, non-steroidal anti-inflammatory drugs (NSAIDs) are being used to treat several types of cancer. Overexpression of cPLA_2_ has been observed in several human cancers and cell lines such as NSCLC (non small lung squamous carcinomas) (Heasley et al. 1997) or human colorectal adenocarcinomas (Osterstrom et al. 2002). Down-regulation of this enzyme by deleting the Pla2g4 locus can cause a reduction in the size of these tumors (Hong et al. 2001); furthermore, blockage of cPLA_2_ activation through inhibition of C1P formation might have similar effects as knocking down cPLA_2_, thereby causing a reduction of arachidonic acid release and prostanoid formation. Otherwise, although some SK inhibitors are known to cause partial inhibition of CERK when used at high concentrations, effective and specific inhibitors of CERK have not been reported until recently (Kim et al. 2005). The latter authors have developed some analogs of the SK inhibitor F-12509A and found an olefin isomer with promising applications as it inhibits CERK without affecting SK or diacylglycerol kinase activities.

## Concluding Remarks

Many sphingolipids, in particular ceramides and their derivatives C1P and S1P, are bioeffector molecules that control important cellular processes, some of which are directly implicated in carcinogenesis. Inflammation is one of the processes that have been associated to tumor formation for many years. The inflammatory response is a complex process that involves hundreds of genes; hence, there are many genes in the inflammatory pathways that might contribute to the development of cancer. Some of these pathways, as well as pathways involved in the regulation of cell proliferation and apoptosis are regulated by sphingolipids. Therefore, sphingolipid metabolism may be an appropriate area for identification of therapeutic targets. Cancer chemotherapy has classically focused on targeting DNA or key proteins that are involved in DNA synthesis and repair with the aim of causing lethal damage to malignant cells with tolerable toxicity to normal tissues. In the last few years, cancer drug development has also been based on new observations related to sphingolipid metabolism aiming at designing more selective approaches for inducing tumor cell death or cytostasis. Manipulation of ceramide, S1P and C1P levels, or the activity of the enzymes that control their metabolism might provide a unique opportunity for cancer treatment or prevention.
